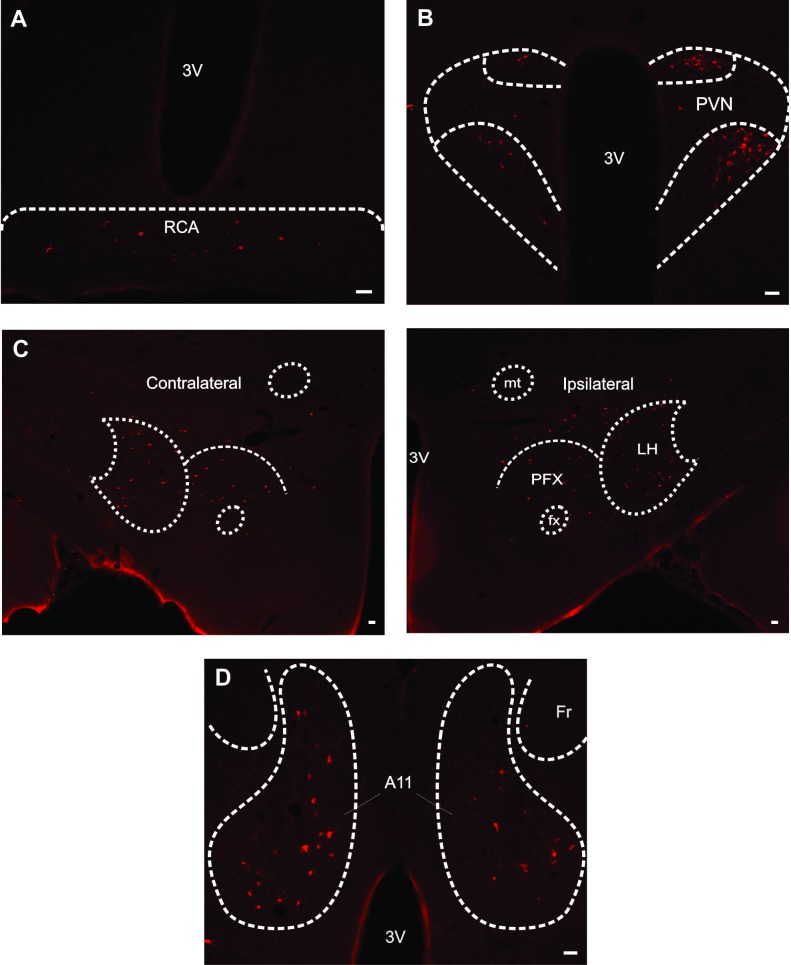# Correction: Bilateral Descending Hypothalamic Projections to the Spinal Trigeminal Nucleus Caudalis in Rats

**DOI:** 10.1371/annotation/7c794f90-1101-4196-8b10-4e3e320a7aac

**Published:** 2013-09-27

**Authors:** Khaled Abdallah, Alain Artola, Lénaic Monconduit, Radhouane Dallel, Philippe Luccarini

Due to issues with the typesetting process, there were error in Figures 1,3, and 4. Correct versions of these Figures are available below.

Figure 1: 

**Figure:**
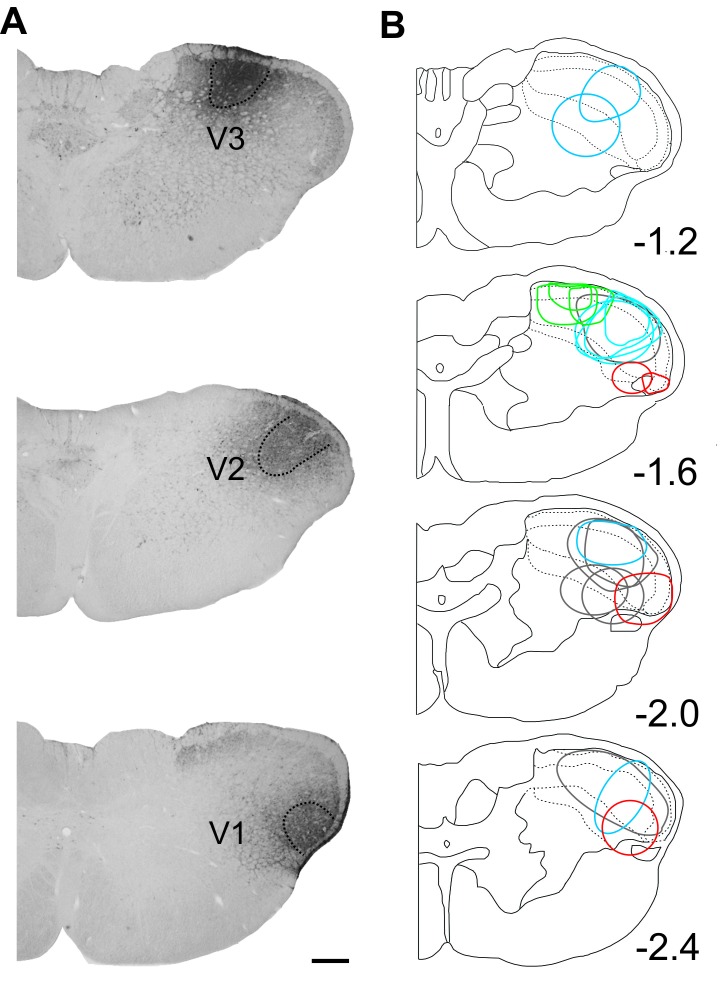


Figure 3: 

**Figure:**
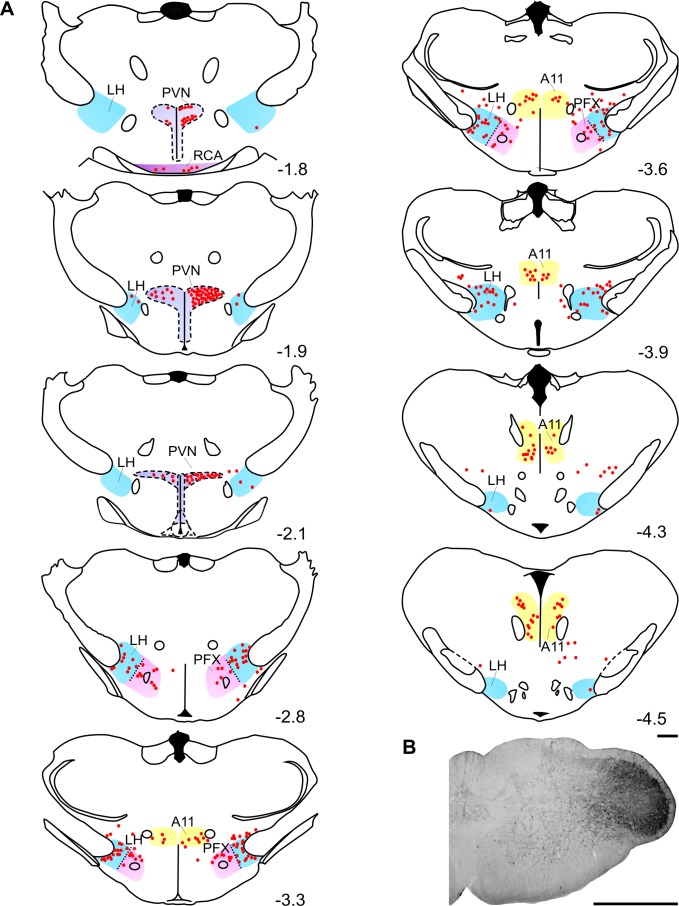


Figure 4: 

**Figure:**